# Mechanical signal-chromatin interactions: molecular networks from nuclear membrane force transmission to epigenetic regulation

**DOI:** 10.3389/fmed.2025.1631645

**Published:** 2025-10-09

**Authors:** Shili Yang, Huaiquan Liu, Bo Chen, Haiyang Kou, Lingyan Lai, Xinyan Zhang, Yunling Xu, Yu Sun

**Affiliations:** Guizhou University of Traditional Chinese Medicine, Guiyang, Guizhou, China

**Keywords:** mechanical force, chromatin, mechanotransduction, cytoskeleton, LINC complex, nuclear envelope, epigenetic regulation

## Abstract

Cells transmit extracellular physical signals across the membrane into the nucleus through membrane mechanoreceptors (such as integrins, mechanically gated ion channels) and the cytoskeletal network. This process leads to redistribution of nuclear membrane tension and dynamic adjustment of chromatin conformation. This process is a core mechanism for cells to sense the microenvironment and regulate physiological activities. As a key hub for mechanotransduction, the linker of nucleoskeleton and cytoskeleton (LINC) complex cooperates with nuclear lamins through the interaction of SAD1/UNC84 domain containing protein (SUN)-Klarsicht, ANC-1 and Syne homology (KASH) domain proteins. Together, they establish a mechanical conduction pathway across the nuclear membrane, mediating the precise transmission of mechanical signals into the nucleus. This then regulates chromatin spatial arrangement and epigenetic modifications. This review systematically analyzes the transmembrane transduction mechanisms of mechanical stimuli (integrin-focal adhesion signaling axis, force-induced activation of Piezo/Transient Receptor Potential Vanilloid (TRPV) family channels, signal integration by primary cilia). It clarifies the rules for force transmission into the nucleus via the cytoskeleton-LINC complex. It reveals the regulatory effects of mechanical force on chromatin three-dimensional topological remodeling and epigenetic modifications. It focuses on organizing the molecular network of the “mechanical stimulus-structural remodeling-epigenetic regulation” cascade. This article aims to provide a theoretical framework for a deeper understanding of the role of mechanical-epigenetic coupling in tissue development and disease progression. It also offers a systematic reference for research in related fields.

## 1 Introduction

In complex biological microenvironments, cells dynamically regulate their physiological activities by sensing mechanical stimuli. This process relies on a multi-level signal transduction system from the plasma membrane to the nucleus. Recent studies show (1, 2) that mechanical stimuli not only regulate fundamental biological processes like cell migration and proliferation, but also remodel chromatin three-dimensional conformation through nuclear membrane force transmission mechanisms. This establishes a dynamic link between mechanical signals and epigenetic modifications, providing new perspectives for revealing the role of mechanical-epigenetic coupling in tissue development and pathological processes. As the core hub for mechanical signal transduction, the nuclear envelope determines the efficiency of nuclear transduction of mechanical stimuli through the dynamic regulation of its molecular components. Studies indicate (3) that the LINC complex, formed by SUN domain-KASH domain interactions, spans the nuclear envelope to form a physical bridge. It mediates the mechanical coupling between cytoskeletal actin filaments and the lamin A/C network, establishing a mechanical conduction pathway across the nuclear envelope. After extracellular mechanical stimuli are transmitted to the nuclear envelope via integrin-mediated focal adhesion systems, Piezo ion channels, TRPV ion channels, or primary cilia sensing systems, they can trigger phosphorylation modifications of lamin proteins. This changes nuclear membrane tension and remodels chromatin anchoring sites (4). Under external tensile force, the LINC complex can promote the dissociation of emerin protein from the nuclear envelope, releasing its constraint on heterochromatin regions marked by H3K9me3, thereby enhancing chromatin accessibility (5). At the same time, mechanosensitive channels Piezo1 and TRPV4 regulate calcium signaling and the Yes-associated protein (YAP)/Transcriptional coactivator with PDZ-binding motif (TAZ) signaling pathway. They cooperate with chromatin topological remodeling to achieve mechanical programming of gene expression (6). Importantly, changes in matrix stiffness can reshape the distribution of chromatin open regions detected by ATAC-Seq. This significantly increases the accessibility of YAP target gene promoter regions and is accompanied by upregulation of H3K27ac modification levels (7). These findings collectively reveal the cascade mechanism of “mechanical stimulus-structural remodeling-epigenetic regulation.” They provide a theoretical framework for understanding the conversion of physical signals into epigenetic memory.

## 2 Mechanical force sensing: molecular mechanisms of transmembrane sensors

### 2.1 Integrin-focal adhesion signaling axis

Integrins are a core family of transmembrane receptors for cell adhesion. They are formed by non-covalent interactions between 18 α subunits and 8 β subunits, resulting in 24 types of heterodimers (8). These transmembrane proteins recognize extracellular matrix (ECM) components, pathogen surface antigens, and ligands on adjacent cell surfaces via their extracellular domains. Simultaneously, they establish mechanical connections to the cytoskeleton through their intracellular domains, forming the molecular basis for microenvironment sensing. Research finds (9) that integrins exist in three typical conformational states: inactive folded state, intermediate extended state, and highly activated state. In the resting state, the genu domain of the α subunit and the I-EGF1~2 region of the β subunit form a tightly bent conformation, with the intracellular segment closed. When subjected to mechanical stimuli, their leg domains undergo upright reconstruction. This causes the head domains of the α/β subunits to move away from the plasma membrane, forming an extended conformation. As activation progresses, hybrid domain splaying triggers a “closed-open” transition of the head domain, while the intracellular domains dissociate (10). Activated integrins anchor ECM components like collagen or fibronectin via their extracellular domains. Their intracellular segments couple to the actin cytoskeleton via talin and vinculin, forming focal adhesion complexes with mechanosensing functions. Importantly, mechanical force stimulation can induce the formation of nanoscale integrin clusters on the membrane surface. This promotes the recruitment and maturation of focal adhesion kinase (FAK) by enhancing binding affinity to talin (11), thereby enabling bidirectional transduction of mechanical and biochemical signals (12). Further studies show (13, 14) that mechanical regulation by ECM stiffness prolongs the stability of integrin clusters. This enhances the duration of phosphorylation at Tyr397 of FAK and significantly improves the efficiency of mechanical signal transmission into the cell. Under mechanical stress, changes in the expression of different integrin subtypes are highly specific. Their regulatory mechanisms include nanoscale conformational rearrangement, subtype-specific signaling pathways, and epigenetic programming. Experiments using a three-dimensional optogenetic molecular force platform confirmed that applying mechanical stimulation of 1Hz/20pN to Hey ovarian cancer cell spheroids selectively activated αvβ3 integrin (expression increased 2.8 times). Applying 0.5Hz/10pN stimulation preferentially induced membrane localization of αvβ6 integrin (increased 3.2 times). This frequency- and amplitude-dependent subtype differentiation originates from the unique force-induced conformational change in the hinge region of the β3 subunit. Full-atom molecular dynamics simulations showed its tension response threshold is 4.3pN lower than that of β6 (15). Furthermore, different types of mechanical force environments (such as tensile stress and compressive stress) also significantly affect integrin expression. Zhu et al. (16) applied dynamic tensile/compressive stress (1,000–4,000 μstrain, 0–12 h) to human periodontal ligament fibroblasts using a four-point bending device. Real-time quantitative PCR results showed that integrin β1 mRNA expression was downregulated in a stress-dependent manner. The degree of downregulation was related to stress intensity, type, and duration: after 12 h of 4,000 μstrain stress, the inhibition was most significant, and compressive stress caused a stronger inhibitory effect than tensile stress under the same conditions. This suggests that integrin β1 may act as a mechanical sensor, participating in adaptive cell remodeling by differentially responding to stress types. In weightlessness or microgravity environments, integrin expression shows comprehensive downregulation. Zhi et al. (17) treated rat calvarial osteoblasts using a clinostat to simulate weightlessness. They found that mRNA and protein expression of integrin α5, αv, and β1 subunits decreased over time. Specifically, α5 mRNA decreased by 11.3% after 24 h and 18.7% after 48 h of weightlessness; β1 protein decreased by 27.5% after 72 h of weightlessness. This general downregulation weakens cell adhesion to the extracellular matrix, confirming that the mechanical force application environment specifically affects integrin expression.

### 2.2 Mechanically gated ion channels

Mechanically gated ion channels are a class of transmembrane proteins that open or close in response to changes in mechanical stress on the cell membrane. Their gating mechanism primarily relies on tension, shear force, or curvature changes within the lipid bilayer to convert mechanical stimuli into ion flow. This process does not depend on classic ligand-receptor interactions, enabling rapid sensing and response to mechanical changes in the cellular microenvironment (18). In mammalian systems, the Piezo and TRPV family channels play key roles in the nuclear transmission of mechanical signals and epigenetic regulation due to their unique mechanical-electrochemical coupling properties. Among them, the Piezo family consists of Piezo1/Piezo2 subunits. They achieve mechanical gating through the synergistic action of the lipid membrane tension model and the tether tension model. In the lipid membrane tension model, the channel paddle region bends at rest, forming a “nano-bowl” (diameter 24 nm, depth 9 nm), increasing local membrane curvature. Mechanical tension causes the paddles to flatten, the nano-bowl unfolds, leading to membrane area expansion (~250 nm^2^). The stored elastic potential energy then drives the opening of the transmembrane pore gate (19). The tether tension model mainly relies on the binding of the E-Cadherin extracellular region to the Cap domain of Piezo1 and the connection of its intracellular end to the β-catenin-vinculin-F-actin complex. This transmits cytoskeletal tension to the channel, cooperatively regulating pore opening (19). During gating, conformational changes in the peripheral paddles are transmitted to the central pore region via a long beam structure (beam, ~90 Å). The beam acts as a lever with L1342/L1345 as the fulcrum, converting the large displacement of the paddles into subtle deformation in the pore region, thereby amplifying mechanical force and enabling precise gating (20). Piezo channels are non-selective cation channels, primarily mediating transmembrane transport of sodium ions (Na^+^), potassium ions (K^+^), and calcium ions (Ca^2+^), with Ca^2+^ influx being particularly crucial in signal transduction (21). Their ion permeability characteristics are regulated by key amino acid sites in the pore region. For example, acidic residues (like Glu2493) in the Piezo1 pore region enhance cation selectivity through electrostatic interactions, while hydrophobic residues influence single-channel conductance and pore block characteristics (21). When the channel is activated by mechanical force, Ca^2+^ influx triggers downstream signaling cascades (such as the calmodulin kinase CaMKII pathway), thereby regulating physiological processes like cell migration and gene expression (22, 23). Importantly, this channel forms a mechanical coupling system with the filamentous actin (F-actin) network via the Epithelial cadherin (E-cadherin)/beta-catenin (β-catenin) complex. This allows mechanical energy transmitted by the cytoskeleton to be precisely concentrated on the force-sensitive regions of the channel, achieving mechanotransduction at the subcellular scale (24, 25). Studies confirm (26, 27) that in non-neuronal cells like vascular endothelial cells and erythrocytes, Piezo1 dominates the perception of blood flow shear stress. It maintains circulatory homeostasis by regulating vascular tension and nitric oxide secretion, while also participating in osmolarity-dependent erythrocyte shape regulation. In dorsal root ganglia and trigeminal ganglia, Piezo2 is specifically expressed in mechanosensitive neurons, responsible for transmitting neural signals like touch, vibration, and proprioception. It is important to emphasize that the activation thresholds of the two channel types differ: Piezo1 requires higher intensity, sustained mechanical stimuli, suitable for detecting steady-state mechanical signals; Piezo2, however, responds rapidly to transient weak stimuli, and its fast inactivation kinetics highly match the transient signal transmission needs of the nervous system.

TRPV, as an important mediator of multimodal signal perception, includes six subtypes (TRPV1-6). Among them, TRPV1-4 subtypes play a central role in mechanical signal detection. These non-selective cation channels integrate stimuli from multiple physical fields, such as mechanical stress, temperature fluctuations, and osmotic pressure changes, to achieve transmembrane transduction of extracellular environmental information. Research shows (28) that TRPV1 can be activated not only by noxious heat but also responds to mechanical tension and osmotic pressure changes; TRPV2 exhibits dual sensitivity to hypotonic environments and mechanical stress (29); TRPV4, as a typical mechanosensitive channel, can directly decode membrane tension gradients. It triggers calcium influx by sensing fluid shear stress, regulating downstream mechanical signaling networks (30). Structurally, the S1–S4 transmembrane region of TRPV4 forms a dynamic paddle-like domain highly responsive to membrane stretch. When the agonist GSK101 binds to the intracellular binding pocket formed by the S1–S4 domains, it stabilizes the conformational rearrangement of the S4–S5 linker, thereby driving channel opening (31), completing the mechanical signal transduction pathway.

### 2.3 Mechanotransduction by primary cilia

Primary cilia are ubiquitous non-motile sensory organelles on the surface of eukaryotic cells. Their “9+0” axoneme structure, composed of microtubules (distinguished from the “9+2” pattern of motile cilia), provides the core structural basis for mechanical signal perception (32). This structure is widely distributed on mammalian cell surfaces and can integrate mechanical stimuli and chemical signals to regulate cellular physiological activities, hence termed the “cellular antenna” (33). The ciliary membrane harbors various mechanosensitive receptors (such as TRPV4, polycystin complex PC1/PC2, and GPCRs). It utilizes the intraflagellar transport (IFT) system for bidirectional transport of signaling molecules: the IFT-B complex drives anterograde transport (from the basal body to the tip), and the IFT-A complex mediates retrograde transport (from the tip to the basal body). This process relies on motor proteins like kinesin Kif3a, ensuring precise localization and activation of mechanical signal effector molecules (34, 35). When external mechanical stimuli (such as fluid shear force or compressive stress) act on the primary cilium, the cilium bends, altering membrane tension. This triggers TRPV4 channel opening and causes local Ca^2+^ influx, activating downstream AC6-cAMP-PKA/COX2 pathways. Simultaneously, mechanical deformation promotes the migration of receptors like Parathyroid Hormone 1 Receptor (PTH1R) to the cilium. By regulating the TGF-β/BMP-Smad and Hedgehog (Hh) signaling cascades, mechanical signals are converted into biochemical responses (34–36). In the Hh pathway, the primary cilium acts as a dynamic signaling platform: at rest, Ptch1 inhibits Smo from entering the cilium; upon mechanical stimulation, Smo translocates to the ciliary tip, relieving inhibition of Gli transcription factors and promoting target gene expression, thereby regulating cell differentiation and matrix remodeling (27, 30). Studies show that the IFT mechanism dependent on Intraflagellar Transport 88 (IFT88) plays a decisive role in maintaining ciliary structural stability and mechanical signal transduction efficiency. Gene knockout experiments show that IFT88 deficiency severely impairs the transmembrane transduction ability of mechanical signals (37). Further research finds that increasing ciliary length reduces its bending stiffness. This dynamic morphological regulation can significantly improve signal transduction efficiency (38), indicating that mechanical adaptive adjustment of the cilium is a fundamental basis for its mechanosensory function. Research also found (35) that in the skeletal system, primary cilia of bone marrow mesenchymal stem cells (BMSCs) promote osteogenic differentiation by mediating fluid shear force-responsive TGF-β1/BMP-Smad signaling. IFT88 silencing significantly inhibits this process and reduces BMP-Smad signaling activity. Additionally, mechanical modeling studies show that the mechanosensitivity of primary cilia is regulated by their morphological parameters: increased length reduces flexural rigidity and expands the stress influence zone, while diameter changes affect bending resistance by altering the cross-sectional moment of inertia. This allows cells to dynamically adjust mechanical signal perception sensitivity (39, 40). Therefore, primary cilia also play an indispensable role in mechanotransduction.

**Figure 1:**
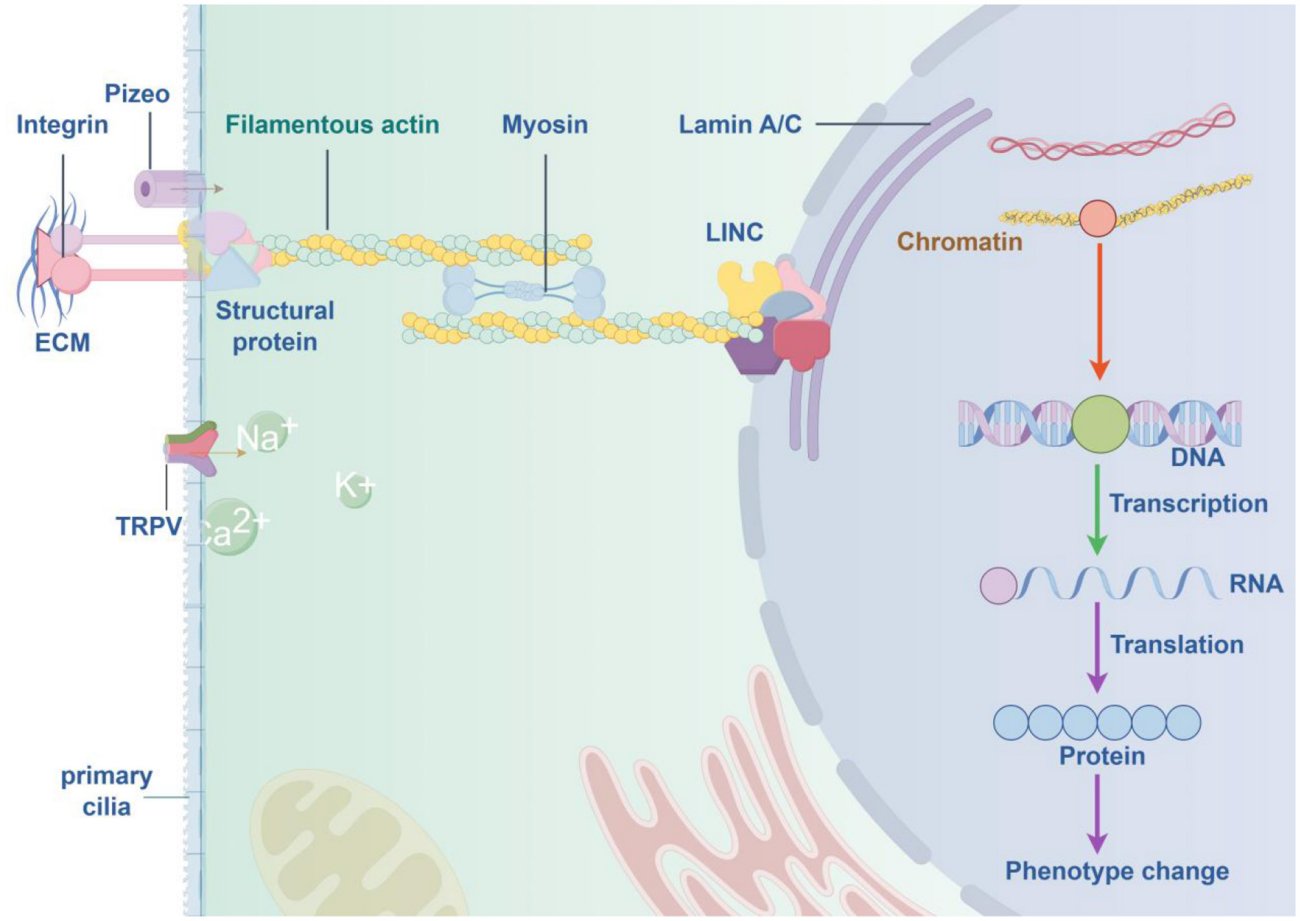
Conceptual diagram of the cascade reaction from mechanical force sensing to chromatin remodeling.

The concepts discussed above are summarized in the following conceptual diagram, see Figure 1 (By Figdraw).

## 3 Intranuclear force transmission: synergistic action of cytoskeleton-LINC complex

### 3.1 Mechanical conduction properties of the cytoskeletal network

The cytoskeletal network, as a key mechanical conduction system in cells, converts external mechanical stimuli into intracellular biochemical signals through dynamic reorganization of actin filaments, microtubules, and intermediate filaments, achieving cross-scale transmission of force-chemical signals. Actin filaments are double-helical structures with a diameter of ~7 nm formed by the polymerization of actin monomers, building a physical bridge for mechanical conduction through integration with the ECM (41). When subjected to mechanical force, actin filaments undergo dynamic changes of first depolymerization and then polymerization, converting mechanical signals into cytoskeletal structural remodeling and further enhancing their mechanical conduction efficiency (42). Microtubules are hollow tubular structures with a diameter of 25 nm assembled from α/β-tubulin heterodimers, and the guanosine triphosphate (GTP) cap structure at their plus end maintains lattice stability through longitudinal tension. Different types of mechanical forces can induce conformational changes at microtubule ends, affecting the polymerization-depolymerization balance. For example, under tension, the GTP hydrolysis rate at microtubule ends decreases, reducing dynamic instability, while pressure promotes GTP hydrolysis, leading to catastrophic depolymerization (43). Microtubules regulate cellular mechanical responses through this dynamic instability mechanism (44). Intermediate filaments, as important components of the cytoskeleton, play a core role in mechanical force-mediated processes through their unique structural properties and dynamic regulation. Their protein family includes vimentin, keratin, nuclear lamins, etc., forming a highly ordered fibrous network in the cytoplasm and nucleus. Intermediate filament monomers consist of a central α-helical rod domain and variable head and tail domains, self-assembling into rope-like fibers with a diameter of approximately 10 nm through tetramerization, possessing both high tensile strength and flexibility, serving as key “shock absorbers” for cells to cope with mechanical stress (45). This structure forms a cross-cellular mechanical signal transmission and integration network by connecting membrane desmosomes, hemidesmosomes, ECM, and the nuclear membrane (45). Actin filaments, microtubules, and intermediate filaments form a composite network through cross-linking proteins such as plectin: microtubules and actin filaments provide dynamic mechanical responses, while intermediate filaments stabilize the overall architecture. Taking fluid shear stress as an example, microtubule-dependent dynein and intermediate filaments work together to generate vertical pulling force, regulating the dynamics of integrin clusters and cell adhesion behavior, while activating the p38 MAPK/JNK pathway to coordinate cytoskeletal remodeling through the Rac1/cdc42/myosin II axis (46).

### 3.2 Structure-function coupling of the LINC complex

The LINC complex is formed by the interaction between inner nuclear membrane SUN domain proteins and outer nuclear membrane KASH domain proteins across the perinuclear space. This forms a bridge connecting the cytoskeleton in the cytoplasm to the nucleoskeleton inside the nucleus. It thereby mediates the transmission of mechanical force from the ECM to the nucleus, regulating processes like nuclear positioning, chromatin spatial conformation, and gene expression (47). SUN domain proteins are localized to the inner nuclear membrane. Their name derives from the 34% similarity of their C-terminal amino acid sequence to yeast Sad1 protein and C. elegans UNC-84 protein. SUN domain proteins are often classified into 5 types. Among them, only SUN1 and SUN2 are widely distributed in various cell types, while SUN3, SUN4, and SUN5 are specifically expressed only in sperm cells (48). SUN domain proteins bind KASH from both their C- and N-termini to complete the connection from the nuclear lamina to the cytoskeleton. SUN1 primarily acts on the microtubule cytoskeleton, regulating microtubule dynamics, and actomyosin contractility. SUN1 deficiency accelerates microtubule depolymerization and weakens microtubule-mediated force transmission (49). SUN2 primarily regulates the level of cytoplasmic actin, the main structural component of microfilaments (50). SUN2 depletion typically leads to reduced F-actin polymerization, nuclear blebbing, and telomere dislocation, diminishing the degree of cytoskeletal force transmission across the membrane (51). KASH domain proteins are localized to the outer nuclear membrane. Their name derives from the homology of their C-terminal transmembrane region amino acid sequence (e.g., human Syne-1, Syne-2 C-terminal sequences) to Drosophila Klarsicht protein and C. elegans ANC-1 protein. KASH domain proteins are often classified into 6 types: Nesprin1, Nesprin2, Nesprin3, Nesprin4, KASH5, and Lymphoid-restricted membrane protein (Jaw1/LRMP) (52). Nesprin proteins consist of a C-terminal KASH domain, a series of spectrin repeat domains, and an actin-binding domain. Nesprins are typical KASH proteins; their C-terminus inserts into the nuclear membrane bilayer and binds to the SUN domain, forming the SUN-KASH complex spanning the nuclear envelope. Nesprin1 consists of the large isoform Nesprin-1G and the small isoforms Nesprin-1α, Nesprin-1β. Nesprin2 similarly consists of Nesprin-2G and the small isoforms Nesprin-2α, Nesprin-2β, Nesprin-2γ. Nesprin1 and Nesprin2 share high homology, are highly elastic, and are separated at the C-terminus by helical small molecule sequences. They can interact with actin through multiple pathways, effectively transmitting mechanical force between the cytoskeleton and the nucleus (53). Nesprin3 consists of Nesprin-3α and Nesprin-3β. It can bridge the nucleoskeleton and cytoskeleton, participating in intracellular structure positioning and migration (54). Nesprin4 has a simpler structure, consisting of only one spectrin repeat and a KASH domain, lacking an actin-binding domain. It functions with cytoplasmic microtubules, mediating mechanical force transmission (55). KASH5 is a meiosis-specific coiled-coil protein. It activates dynein adaptors, promoting microtubule mechanical force transmission to meiotic chromosomes, and plays an important role in their synapsis and fertility (56). LRMP (Jaw1) is a membrane protein localized to the endoplasmic reticulum and outer nuclear membrane. It plays an important role in nuclear positioning by interacting with SUN proteins and microtubules (57). The SUN-KASH interaction depends on two adjacent SUN domains within the SUN trimer, which together form the KASH domain binding pocket (58). Studies confirm that differences in KASH domain length affect the strength of SUN-KASH interactions. Longer KASH domains can bear greater mechanical force (59). Other studies show that when SUN-KASH structures form higher-order 6:6 complexes, they can enhance the elasticity of the LINC complex against mechanical tension (60). The physical connection between SUN and Nesprins allows the LINC complex to transmit force directly into the nucleus and further to various proteins interacting with SUN, including nuclear lamins.

### 3.3 Mechanical mediation by nuclear lamins

Lamins, as core structural components of the nuclear lamina in eukaryotic cells, belong to the type V intermediate filament protein family. They build a fibrous network beneath the inner nuclear membrane, not only providing mechanical stability to the nucleus but also deeply involved in chromatin organization, gene regulation, and signal transduction (61). Their structure comprises three functional domains: a head region responsible for anchoring to the inner nuclear membrane, an α-helical rod domain forming the structural scaffold, and a tail domain mediating chromatin attachment. Together, they constitute a key hub for mechanical force transmission into the nucleus (62). Nuclear lamins mainly include A-type lamins (lamin A, lamin C, and minor isoforms Aδ10, Aδ50, C2) encoded by the LMNA gene, and B-type lamins (lamin B1, lamin B2, lamin B3) encoded by the LMNB1 and LMNB2 genes (63–65). Compared to loss of LMNA/C or LMNB1, deletion of LMNB2 has a lesser impact on nuclear lamina structure. Lamins provide the nucleus with mechanical stiffness crucial for normal cell physiology, resisting mechanical force-induced nuclear deformation and genomic perturbation (66, 67). Lamin A/C proteins play key roles in maintaining nuclear envelope mechanical stability, regulating chromatin organization, mediating gene expression programs, and participating in cell differentiation processes (68). As core components of the nucleoskeleton, Lamin A/C forms a three-dimensional fibrous network structure, endowing the nucleus with mechanical properties to resist external mechanical stress. Simultaneously, it constructs a mechanical transmission pathway across the nuclear envelope via the LINC complex, transmitting extracellular mechanical stimuli into the nucleus. This triggers changes in nuclear envelope morphology and dynamic reorganization of the Lamin A/C network, thereby influencing nuclear shape and chromatin spatial arrangement by regulating nuclear membrane tension (69). For example, in soft viscoelastic matrix environments, downregulation of Lamin A/C expression can induce nuclear envelope wrinkling and increase chromatin accessibility, promoting the activation of reprogramming-related genes like Ascl1 and Oct4 (70). Conversely, loss or mutation of Lamin A/C leads to abnormal nuclear envelope structure, chromatin dynamic imbalance, and increased reactive oxygen species levels. This subsequently induces calcium signaling dysregulation via the SIRT1-CaMKII-RYR2 signaling pathway (68). Molecularly, the C-terminal immunoglobulin-fold domain of Lamin A/C can directly bind nucleosome DNA and form complexes with Barrier-to-Autointegration Factor (BAF). It anchors chromatin through a “nucleosome-bridging” structure, restricting its excessive movement to maintain genomic structural stability (71). Studies show (72) that nuclei lacking lamin A/C exhibit increased deformation rates under tensile stress and decreased cell survival, indicating its irreplaceable role in maintaining the tensile resistance of the nucleoskeleton. In contrast, lamin B1 deficiency causes nuclear envelope blebbing but does not affect overall nuclear stiffness or mechanical stability. This suggests its function is more focused on maintaining nuclear envelope structural integrity than mechanical properties. Lamin B2 has functions similar to lamin B1; both regulate nuclear structure by anchoring chromatin to the nuclear periphery. Lamin B1 deficiency causes lamin-associated domains to detach from the nuclear envelope, leading to chromatin decompaction and increased movement rates. This disrupts the spatial segregation of chromosome territories and A/B compartments, indirectly affecting the nucleus's response to physical stress (73). Furthermore, lamins and intranuclear scaffold proteins form a “tug-of-war model”: lamin B1 provides outward anchoring force at the nuclear periphery, while intranuclear scaffold proteins exert inward pulling force. They maintain chromatin distribution and movement homeostasis through mechanical balance, jointly ensuring nuclear structural integrity under mechanical stimulation (73). However, other studies indicate that lamin B1 plays an important role in maintaining nuclear structural integrity and mediating the anchoring of the nucleus to LINC protein complexes (74, 75), suggesting a potential link to mechanical force transmission.

## 4 Chromatin mechanical response: molecular pathways of epigenetic reprogramming

### 4.1 Chromatin three-dimensional structure and primary functions

The three-dimensional structure of chromatin exhibits a dynamic hierarchical organization from basic to higher-order levels. These include nucleosomes, chromatin fibers, chromatin loops, topologically associating domains (TADs), and compartments. Each level participates in gene expression regulation and cell function differentiation through dynamic remodeling. The nucleosome, as the basic structural unit of chromatin, consists of a histone octamer core (composed of two molecules each of H2A, H2B, H3, and H4) with a diameter of approximately 10 nm. It is wrapped by 147 bp of DNA double helix. Linker histone H1 binds to the DNA entry/exit sites outside the nucleosome core particle, maintaining the stability of higher-order chromatin fiber structures (76). The N-terminal tails of core histones can undergo various post-translational modifications, such as methylation, acetylation, and phosphorylation. By influencing nucleosome-nucleosome interactions and effector protein recruitment, they play key roles in biological processes like chromatin compaction, gene expression regulation, DNA replication, and repair (77). For example, nucleosomes can weaken DNA-histone binding through H3K56 acetylation, increasing the passage efficiency of RNA polymerase II during transcriptional elongation. Nucleosome Assembly Protein 1 (NAP1) influences chromatin openness by regulating nucleosome assembly dynamics (78). Additionally, nucleosomes can undergo local conformational changes in response to ATP-dependent Switch/Sucrose Non-Fermentable Chromatin Remodeling Complex (SWI/SNF), thereby participating in DNA accessibility regulation. The chromatin fiber, as a higher-order structural unit of chromatin, consists of a core of tandem nucleosomes. It folds into an approximately 30 nm fiber structure mediated by linker histone H1/H5, playing an important role in gene expression regulation, DNA replication, and maintaining genomic stability (79). Although the folding pattern of the chromatin fiber has been long debated, recent studies indicate it generally follows a two-start zigzag double helix structure: adjacent nucleosomes form a zigzag arrangement via linker DNA, and two nucleosome chains intertwine to form a double helix. Changes in nucleosome spacing affect fiber flexibility, but local subunits still maintain the two-start configuration (80). This structural plasticity is the core basis for gene expression regulation. When chromatin fibers form tightly compacted heterochromatin structures by enhancing nucleosome-nucleosome interactions, they hinder transcription factor binding and inhibit chromatin loop extrusion mechanisms, thereby suppressing gene transcription (81). Conversely, the normally loose, open chromatin state maintains dynamic equilibrium between nucleosomes, exposing transcription factor binding sites. This promotes their specific binding to DNA and the recruitment/elongation of RNA polymerase II (82). Chromatin loops are key units of three-dimensional higher-order structure in eukaryotic genomes. They form specific spatial domains by folding chromatin fibers, regulating life activities like gene expression, DNA replication, and genomic stability (83). Their core formation mechanism is mediated by the CCCTC-binding factor (CTCF) structural protein and the cohesin complex. They dynamically construct TADs through the “loop extrusion” process, bringing distant regulatory elements and target gene promoters closer in the linear genome for precise transcriptional regulation (84). Studies show that the synergistic action of CTCF and cohesin can form chromatin loops through directional sliding, blocking or promoting distal enhancer-promoter interactions. This process is particularly critical for the stochastic activation of neural development genes (85, 86). Chromatin compartments are important functional units of 3D genome organization. They describe the spatial partitioning of chromatin within the nucleus and its association with gene activity. Based on interaction patterns revealed by Hi-C technology, chromatin compartments are divided into open A compartments (active compartments) and compact B compartments (inactive compartments). These correspond to the spatial distribution of euchromatin and heterochromatin, respectively (87). A compartments are enriched in actively transcribed genes, histone modifications (such as H3K4me3, H3K27ac), and high chromatin accessibility. They are typically located in the nuclear interior, facilitating the recruitment of transcription factors and RNA polymerases. B compartments are characterized by gene silencing, heterochromatin marks (like H3K9me3, H3K27me3), and compact structure. They are mostly distributed in the nuclear periphery, suppressing gene expression (88, 89).

### 4.2 Mechanical force-mediated chromatin functional adjustment and three-dimensional topological remodeling

At the epigenetic level, mechanical force regulation of chromatin primarily manifests as altering chromatin structure and accessibility, thereby regulating gene expression and influencing cell fate reprogramming. External mechanical stimuli are transmitted to the nuclear envelope via microtubules and actin in the cytoskeleton-LINC complex. This triggers conformational changes in the nucleoskeletal protein Lamin A/C, regulating chromatin function and spatial arrangement (90). For example, in rigid matrix environments, unphosphorylated YAP/TAZ continuously enters the nucleus and binds to TEAD transcription factors, activating downstream osteogenic genes like RUNX2. Simultaneously, it mediates changes in H3K9me3 methylation modifications and chromatin conformation adjustment via the LINC complex (90). In studies of chromatin functional regulation mechanisms, experiments show (91) that applying mechanical forces of the same magnitude but different directions (perpendicular or parallel to the cell long axis) produces differential effects: force perpendicular to the long axis causes the greatest chromatin stretch, corresponding to the highest gene expression level; force parallel to the long axis causes the least chromatin stretch and the lowest gene expression level. Importantly, gene expression changes can be detected just 15 s after mechanical signal stimulation. This suggests that this regulation does not depend on cytoplasmic biochemical signaling pathways and can directly induce gene expression. Further research reveals that nuclear proteins HP1 and BAF are key molecules transmitting mechanical signals from the nuclear envelope to chromatin. Knockout of these two proteins interrupts mechanical signal transduction and blocks gene expression, confirming the direct regulatory role of mechanical force on chromatin epigenetics (91). Additionally, mechanical strain treatment can broadly enhance chromatin accessibility in cumulus cells. It effectively repairs transcriptional dysregulation in somatic cell nuclear transfer embryos and significantly improves blastocyst development efficiency (92). This indicates that chromatin remodeling acts as a mediating bridge between cellular responses to mechanical force and reprogramming potential. Regarding chromatin topological structure remodeling, mechanical force primarily influences nucleosome conformation by regulating Imitation Switch chromatin remodeling protein (ISWI) (93, 94). During ATP hydrolysis, ISWI induces rigid-body movement in its motor domain. This converts chemical energy into mechanical pulling force on DNA, causing a 1 bp DNA bulge at the SHL2 position of the nucleosome. This disrupts local DNA-histone interactions and reduces nucleosome stability. This conformational change is transmitted via a “twist propagation” or “loop propagation” model, converting chemical energy into DNA deformation potential energy. This drives nucleosome sliding and induces three-dimensional chromatin structure remodeling. Similarly, nucleosome sliding affects compaction density at the chromatin fiber level. Mechanical force can further enhance this effect by regulating nucleosome spacing and fiber folding patterns. At the chromatin loop level, mechanical force enhances chromatin accessibility by activating the YAP-TEAD pathway. This promotes the dynamic formation and adjustment of chromatin loops. This process is closely related to the loop extrusion mechanism of the cohesin complex. Mechanical signals may influence loop expansion by altering the direction of movement or dwell time of cohesin (92). Studies on nuclear envelope-associated mechanisms show (73) that lamins anchor lamin-associated domains (LADs) of chromatin near the nuclear envelope, providing anchoring force for chromatin intranuclear distribution. Knockout of lamin B1 causes some chromatin near the nuclear envelope to migrate toward the nucleoplasm. This is accompanied by looser chromatin folding and increased movement freedom, disrupting the normal segregation of chromatin territories and A/B compartments, leading to higher-order structural disorder (73). Mechanical force can promote phosphorylation of lamin B1, driving chromatin three-dimensional topological remodeling, thereby regulating its epigenetic state (95).

In summary, key proteins, molecular complexes, and pathways involved in mechanotransduction-related chromatin regulation are shown in Table 1.

**Table 1:** Key proteins, molecular complexes, and pathways involved in mechanotransduction-related chromatin regulation.

**Key proteins**	**Molecular complexes**	**Pathways**
Integrins (αvβ3, αvβ6, β1, etc.)	Integrin-focal adhesion complex (containing talin, vinculin, FAK)	Integrin-focal adhesion signaling axis (transmits mechanical signals via conformational activation, FAK phosphorylation)
Piezo1/2, TRPV1-4	–	Piezo/TRPV channel-mediated calcium signaling pathway (Ca^2^? influx activates CaMKII, YAP/TAZ, etc.)
SUN domain proteins (SUN1, SUN2), KASH domain proteins (Nesprin1–4, etc.)	LINC complex (SUN-KASH complex)	Trans-nuclear envelope mechanical conduction pathway (connects cytoskeleton to nucleoskeleton, transmits mechanical force into the nucleus)
IFT88, PTH1R	Intraflagellar transport complex (IFT-A, IFT-B)	Primary cilium-mediated TGF-β/BMP-Smad pathway, Hedgehog pathway (regulates chromatin-related gene expression via signal molecule transport)
Lamin A/C, Lamin B1	Nuclear lamina-LINC complex-associated structure	Lamin-mediated chromatin anchoring and topological remodeling pathway (regulates chromatin spatial arrangement via phosphorylation modifications)
YAP, TAZ, TEAD	YAP/TAZ-TEAD transcription complex	YAP/TAZ pathway (regulates chromatin open regions and target gene promoter accessibility)
FAK (Focal adhesion kinase)	FAK-integrin-cytoskeleton complex	FAK signaling pathway (enhances mechanical signal transmission efficiency via Tyr397 phosphorylation)
HP1, BAF	HP1-BAF complex	Nuclear envelope-chromatin mechanical signal transmission pathway (mediates direct mechanical regulation of gene expression)
ISWI complex subunits	ISWI chromatin remodeling complex	ISWI-mediated nucleosome sliding pathway (affects chromatin three-dimensional structure remodeling)
Cohesin, CTCF	Cohesin-CTCF complex	Chromatin loop extrusion pathway (regulates dynamic formation of chromatin loops and TADs structure)

## 5 Discussion

In summary, cellular perception and response to mechanical signals involve a multi-level molecular network encompassing transmembrane transduction, intranuclear force transmission, and epigenetic regulation. External mechanical stimuli are precisely captured by transmembrane sensors like the integrin-focal adhesion complex, mechanically gated ion channels (e.g., Piezo family, TRPV family), and primary cilia. They are transmitted to the nuclear envelope via the cytoskeleton (microtubules, actin filaments). Through the synergistic action of the LINC complex (SUN-KASH domain proteins) and nuclear lamins (Lamin A/C, Lamin B1, etc.), mechanical signals are converted into intranuclear biochemical responses. Ultimately, this regulates chromatin three-dimensional conformation and epigenetic modifications. Specifically, integrins form nanoscale clusters through conformational activation (from folded to extended state), activating the FAK signaling pathway and enhancing mechanical signal transmission efficiency into the cell (14, 16). Channels like Piezo1/2 and TRPV4 trigger Ca^2^? influx by sensing membrane tension or shear stress, activating the YAP/TAZ pathway and regulating chromatin topological remodeling (6, 30). Primary cilia transport signaling molecules via the IFT system, mediating mechanical responses of the TGF-β/BMP-Smad and Hh pathways (35, 37). Within the nucleus, the LINC complex acts as the core hub for trans-nuclear envelope mechanical conduction. It connects the cytoskeleton to the nucleoskeleton through SUN-KASH protein interactions, regulating nuclear membrane tension and chromatin anchoring sites (47, 58). Lamin A/C maintains nuclear envelope mechanical stability through conformational reorganization, while lamin B1 influences perinuclear chromatin anchoring and three-dimensional topology through phosphorylation modifications (69, 95). Ultimately, mechanical force changes chromatin accessibility and gene expression patterns by regulating nucleosome sliding (mediated by ISWI complex), chromatin loop dynamics (via Cohesin/CTCF synergy), and histone modifications (such as H3K9me3, H3K27ac). This achieves mechanical programming of cell fate (91, 92). This “mechanical stimulus-structural remodeling-epigenetic regulation” cascade mechanism reveals the coupling laws of physical signals and biochemical signals. It provides a new perspective for understanding the mechanical basis of tissue development, cell differentiation, and disease occurrence (such as tumor metastasis, bone metabolism disorders).

Furthermore, mechanical signal-induced chromatin remodeling and epigenetic regulation are closely linked to normal cell physiology and disease states. In normal physiological processes, mechanical stimuli participate in key processes like cell migration, proliferation, and tissue development by regulating chromatin conformation and epigenetic modifications. For instance, mechanical stimuli can remodel chromatin three-dimensional conformation through nuclear membrane force transmission mechanisms, establishing a dynamic link between mechanical signals and epigenetic modifications. This process provides a mechanical-epigenetic coupling regulatory basis for cell fate decisions during tissue development (1, 2). Changes in matrix stiffness can reshape the distribution of chromatin open regions, increase the accessibility of YAP target gene promoter regions, and be accompanied by upregulation of H3K27ac modification levels. This regulates stem cell differentiation direction and maintains tissue homeostasis (7). In pathological states, dysregulation of mechanical signal-chromatin interactions contributes to the occurrence and development of various diseases. For example, in tumors, mechanical force can promote the recruitment and maturation of focal adhesion kinase through specific activation of integrin subtypes (e.g., αvβ3, αvβ6), enhancing mechanical signal transmission efficiency into the cell. This subsequently activates pro-invasive gene expression through chromatin remodeling, promoting tumor metastasis (15). In laminopathies, loss or mutation of Lamin A/C leads to abnormal nuclear envelope structure and chromatin dynamic imbalance. It induces calcium signaling dysregulation via the SIRT1-CaMKII-RYR2 signaling pathway, closely associated with diseases like cardiomyopathy (68). Additionally, under mechanical stress, altered phosphorylation modifications of lamins can lead to remodeling of chromatin anchoring sites, affecting the stability of epigenetic modifications. This mechanism may participate in the pathological progression of degenerative diseases like progeria syndrome (4, 95). Future research needs to focus on the following directions: first, develop mechanical imaging techniques with high spatiotemporal resolution to enable real-time tracking of nuclear membrane tension changes and chromatin dynamic responses induced by mechanical force. Second, strengthen the investigation of specific molecular targets and signaling nodes through which mechanical force acts on chromatin three-dimensional topology. Finally, through multi-level, multi-sample, multi-dimensional research, analyze the molecular mechanisms of mechanical-epigenetic coupling dysregulation in physiological and pathological states. This will lay the foundation for the prevention, treatment, and design of targeted therapeutic strategies for related diseases. In conclusion, mechanical signals convert physical stimuli into epigenetic memory through multi-layered molecular networks. This process has universal significance in cellular adaptive responses. In-depth analysis of mechanical-epigenetic coupling mechanisms will not only help reveal the physical essence of life activities but also provide innovative ideas for precision medicine and bioengineering research.

## References

[1] Irianto J Xia Y Pfeifer CR Athirasala A Ji J Alvey C. DNA damage follows repair factor depletion and portends genome variation in cancer cells after pore migration. Curr Biol. (2017) 27:210–23. 10.1016/j.cub.2016.11.049 27989676 PMC5262636

[2] Nava MM Miroshnikova YA Biggs LC Whitefield DB Metge F Boucas J. Heterochromatin-driven nuclear softening protects the genome against mechanical stress-induced damage. Cell. (2020) 181:800–17. 10.1016/j.cell.2020.03.052 32302590 PMC7237863

[3] Uzer G Bas G Sen B Xie Z Birks S Olcum M. Sun-mediated mechanical LINC between nucleus and cytoskeleton regulates βcatenin nuclear access. J Biomech. (2018) 74:32–40. 10.1016/j.jbiomech.2018.04.013 29691054 PMC5962429

[4] Stephens AD Liu PZ Banigan EJ Almassalha LM Backman V Adam SA. Chromatin histone modifications and rigidity affect nuclear morphology independent of lamins. Mol Biol Cell. (2018) 29:220–33. 10.1091/mbc.E17-06-0410 29142071 PMC5909933

[5] Le HQ Ghatak S Yeung CY Tellkamp F Günschmann C Dieterich C. Mechanical regulation of transcription controls Polycomb-mediated gene silencing during lineage commitment. Nat Cell Biol. (2016) 18:864–75. 10.1038/ncb3387 27398909

[6] Pathak MM Nourse JL Tran T Hwe J Arulmoli J Le DT. Stretch-activated ion channel Piezo1 directs lineage choice in human neural stem cells. Proc Natl Acad Sci USA. (2014) 111:16148–53. 10.1073/pnas.1409802111 25349416 PMC4234578

[7] Jang M An J Oh SW Lim JY Kim J Choi JK. Matrix stiffness epigenetically regulates the oncogenic activation of the Yes-associated protein in gastric cancer. Nat Biomed Eng. (2021) 5:114–23. 10.1038/s41551-020-00657-x 33288878

[8] Arias-Mejias SM Warda KY Quattrocchi E Alonso-Quinones H Sominidi-Damodaran S Meves A. The role of integrins in melanoma: a review. Int J Dermatol. (2020) 59:525–34. 10.1111/ijd.14850 32157692 PMC7167356

[9] Schürpf T Springer TA. Regulation of integrin affinity on cell surfaces. EMBO J. (2011) 30:4712–27. 10.1038/emboj.2011.333 21946563 PMC3243613

[10] Huang M Lin C Chen J. [Integrin activation, focal adhesion maturation and tumor metastasis]. Sheng Li Xue Bao. (2021) 73:151–9. 10.13294/j.aps.2021.0023 33903877

[11] Carton F Casarella S Di Francesco D Zanella E D'urso A Di Nunno L. Cardiac differentiation promotes focal adhesions assembly through vinculin recruitment. Int J Mol Sci. (2023) 24:2444. 10.3390/ijms24032444 36768766 PMC9916732

[12] Mishra YG Manavathi B. Focal adhesion dynamics in cellular function and disease. Cell Signal. (2021) 85:110046. 10.1016/j.cellsig.2021.110046 34004332

[13] Xia M Wu M Li Y Liu Y Jia G Lou Y. Varying mechanical forces drive sensory epithelium formation. Sci Adv. (2023) 9:eadf2664. 10.1126/sciadv.adf2664 37922362 PMC10624343

[14] Cheng B Wan W Huang G Li Y Genin GM Mofrad MRK. Nanoscale integrin cluster dynamics controls cellular mechanosensing via FAKY397 phosphorylation. Sci Adv. (2020) 6:eaax1909. 10.1126/sciadv.aax1909 32181337 PMC7056303

[15] Li B Fu Q Lu Y Chen C Zhao Y Zhao Y. 3D hydrogel platform with macromolecular actuators for precisely controlled mechanical forces on cancer cell migration. Nat Commun. (2025) 16:4831. 10.1038/s41467-025-60062-3 40413192 PMC12103621

[16] Zhu QD Chao YL Chen XM Zhao J. [Regulation of integrin beta1 mRNA expression by mechanical stress in human periodontal ligament fibroblasts]. Hua Xi Kou Qiang Yi Xue Za Zhi. (2008) 26:194–7. 18605464

[17] Yang Z Wang B Li Y Nie J-L Sun X-Q Zhang S. Effects of simulated weightlessness on the expression of integrin subunits in osteoblasts. J. Air Force Med Univ. (2006) 1140–3.

[18] Zeng Z Chen E Xue J. Emerging roles of mechanically activated ion channels in autoimmune disease. Autoimmun Rev. (2025) 24:103813. 10.1016/j.autrev.2025.103813 40194731

[19] Jiang Y Yang X Jiang J Xiao B. Structural designs and mechanogating mechanisms of the mechanosensitive piezo channels. Trends Biochem Sci. (2021) 46:472–88. 10.1016/j.tibs.2021.01.008 33610426

[20] Zhao Q Zhou H Chi S Wang Y Wang J Geng J. Structure and mechanogating mechanism of the Piezo1 channel. Nature. (2018) 554:487–92. 10.1038/nature25743 29469092

[21] Zhao Q Wu K Geng J Chi S Wang Y Zhi P. Ion permeation and mechanotransduction mechanisms of mechanosensitive piezo channels. Neuron. (2016) 89:1248–63. 10.1016/j.neuron.2016.01.046 26924440

[22] Jiang F Yin K Wu K Zhang M Wang S Cheng H. The mechanosensitive Piezo1 channel mediates heart mechano-chemo transduction. Nat Commun. (2021) 12:869. 10.1038/s41467-021-21178-4 33558521 PMC7870949

[23] Wang J Jing F Zhao Y You Z Zhang A Qin S. Piezo1: structural pharmacology and mechanotransduction mechanisms. Trends Pharmacol Sci. (2025). 10.1016/j.tips.2025.06.009 40750459

[24] Ridone P Vassalli M Martinac B. Piezo1 mechanosensitive channels: what are they and why are they important. Biophys Rev. (2019) 11:795–805. 10.1007/s12551-019-00584-5 31494839 PMC6815293

[25] Wang J Jiang J Yang X Zhou G Wang L Xiao B. Tethering Piezo channels to the actin cytoskeleton for mechanogating via the cadherin-β-catenin mechanotransduction complex. Cell Rep. (2022) 38:110342. 10.1016/j.celrep.2022.110342 35139384

[26] Coste B Mathur J Schmidt M Earley TJ Ranade S Petrus MJ. Piezo1 and Piezo2 are essential components of distinct mechanically activated cation channels. Science. (2010) 330:55–60. 10.1126/science.1193270 20813920 PMC3062430

[27] Ranade SS Woo S Dubin AE Moshourab RA Wetzel C Petrus M. Piezo2 is the major transducer of mechanical forces for touch sensation in mice. Nature. (2014) 516:121–5. 10.1038/nature13980 25471886 PMC4380172

[28] Aguettaz E Bois P Cognard C Sebille S. Stretch-activated TRPV2 channels: role in mediating cardiopathies. Prog Biophys Mol Biol. (2017) 130:273–80. 10.1016/j.pbiomolbio.2017.05.007 28546113

[29] Katanosaka Y Iwasaki K Ujihara Y Takatsu S Nishitsuji K Kanagawa M. TRPV2 is critical for the maintenance of cardiac structure and function in mice. Nat Commun. (2014) 5:3932. 10.1038/ncomms4932 24874017 PMC4050274

[30] O'Conor CJ Leddy HA Benefield HC Guilak F. TRPV4-mediated mechanotransduction regulates the metabolic response of chondrocytes to dynamic loading. Proc Natl Acad Sci USA. (2014) 111:1316–21. 10.1073/pnas.1319569111 24474754 PMC3910592

[31] Zhen W Zhao Z Chang S Chen X Wan Y Yang F. Structural basis of ligand activation and inhibition in a mammalian TRPV4 ion channel. Cell Discov. (2023) 9:70. 10.1038/s41421-023-00579-3 37429860 PMC10333285

[32] Gui M Ma M Sze-Tu E Wang X Koh F Zhong ED. Structures of radial spokes and associated complexes important for ciliary motility. Nat Struct Mol Biol. (2021) 28:29–37. 10.1038/s41594-020-00530-0 33318703 PMC7855293

[33] Spasic M Jacobs CR. Primary cilia: cell and molecular mechanosensors directing whole tissue function. Semin Cell Dev Biol. (2017) 71:42–52. 10.1016/j.semcdb.2017.08.036 28843978 PMC5922257

[34] Shuang Z Tingting Z. Recent advances on primary cilia in stem cell differentiation and tooth development. J Oral Maxillofacial Surg. (2019) 29:230–4.

[35] Ti H Zhang Z Yan X Hu H Zhang K Shi S. Primary cilia as mechanosensors in musculoskeletal homeostasis and disease. Pharmacol Res. (2025) 219:107887. 10.1016/j.phrs.2025.107887 40738397

[36] Ruhlen R Marberry K. The chondrocyte primary cilium. Osteoarthritis Cartilage. (2014) 22:1071–6. 10.1016/j.joca.2014.05.011 24879961

[37] Quadri N Upadhyai P. Primary cilia in skeletal development and disease. Exp Cell Res. (2023) 431:113751. 10.1016/j.yexcr.2023.113751 37574037

[38] McIntyre JC Davis EE Joiner A Williams CL Tsai I-C Jenkins PM. Gene therapy rescues cilia defects and restores olfactory function in a mammalian ciliopathy model. Nat Med. (2012) 18:1423–8. 10.1038/nm.2860 22941275 PMC3645984

[39] Spasic M Jacobs CR. Lengthening primary cilia enhances cellular mechanosensitivity. Eur Cell Mater. (2017) 33:158–68. 10.22203/eCM.v033a12 28217833 PMC5922258

[40] Chaoxin L Xiaogang W Yuqin S Yingze Q Wangping D Meizhen Z. Mechanotransduction of the cell and its primary cilium in the microfluidic channel. Chin J Theor Appl Mech. (2021) 53:260–77. 10.6052/0459-1879-20-283

[41] Ross TD Coon BG Yun S Baeyens N Tanaka K Ouyang M. Integrins in mechanotransduction. Curr Opin Cell Biol. (2013) 25:613–8. 10.1016/j.ceb.2013.05.006 23797029 PMC3757118

[42] Cronin NM DeMali KA. Dynamics of the actin cytoskeleton at adhesion complexes. Biology. (2021) 11:52. 10.3390/biology11010052 35053050 PMC8773209

[43] Gudimchuk NB McIntosh JR. Regulation of microtubule dynamics, mechanics and function through the growing tip. Nat Rev Mol Cell Biol. (2021) 22:777–95. 10.1038/s41580-021-00399-x 34408299

[44] Kabir AMR Kakugo A. A new approach to explore the mechanoresponsiveness of microtubules and its application in studying dynamic soft interfaces. Polym J (2021) 53:299–308. 10.1038/s41428-020-00415-5

[45] Kechagia Z Eibauer M Medalia O. Structural determinants of intermediate filament mechanics. Curr Opin Cell Biol. (2024) 89:102375. 10.1016/j.ceb.2024.102375 38850681

[46] Kalli M Li R Mills GB Stylianopoulos T Zervantonakis IK. Mechanical stress signaling in pancreatic cancer cells triggers p38 MAPK- and JNK-dependent cytoskeleton remodeling and promotes cell migration via Rac1/cdc42/Myosin II. Mol Cancer Res. (2022) 20:485–97. 10.1158/1541-7786.MCR-21-0266 34782370 PMC8898300

[47] Janota CS Calero-Cuenca FJ Gomes ER. The role of the cell nucleus in mechanotransduction. Curr Opin Cell Biol. (2020) 63:204–11. 10.1016/j.ceb.2020.03.001 32361559

[48] McGillivary RM Starr DA Luxton GWG. Building and breaking mechanical bridges between the nucleus and cytoskeleton: regulation of LINC complex assembly and disassembly. Curr Opin Cell Biol. (2023) 85:102260. 10.1016/j.ceb.2023.102260 37857179 PMC10859145

[49] Buglak DB Bougaran P Kulikauskas MR Liu Z Monaghan-Benson E Gold AL. Nuclear SUN1 stabilizes endothelial cell junctions via microtubules to regulate blood vessel formation. Elife. (2023) 12:e83652. 10.7554/eLife.83652 36989130 PMC10059686

[50] Sharma R Hetzer MW. Disulfide bond in SUN2 regulates dynamic remodeling of LINC complexes at the nuclear envelope. Life Sci Alliance. (2023) 6:e202302031. 10.26508/lsa.202302031 37188462 PMC10193101

[51] Yue X Cui J Sun Z Liu L Li Y Shao L. Nuclear softening mediated by Sun2 suppression delays mechanical stress-induced cellular senescence. Cell Death Discov. (2023) 9:167. 10.1038/s41420-023-01467-1 37198162 PMC10192198

[52] Jahed Z Domkam N Ornowski J Yerima G Mofrad MRK. Molecular models of LINC complex assembly at the nuclear envelope. J Cell Sci. (2021) 134:e202302031. 10.1242/jcs.258194 34152389

[53] Rajgor D Shanahan CM. Nesprins: from the nuclear envelope and beyond. Expert Rev Mol Med. (2013) 15:e5. 10.1017/erm.2013.6 23830188 PMC3733404

[54] Liao L Qu R Ouang J Dai J. A glance at the nuclear envelope spectrin repeat protein 3. Biomed Res Int. (2019) 2019:1651805. 10.1155/2019/1651805 31828088 PMC6886330

[55] Taiber S Gozlan O Cohen R Andrade LR Gregory EF Starr DA. A Nesprin-4/kinesin-1 cargo model for nuclear positioning in cochlear outer hair cells. Front Cell Dev Biol. (2022) 10:974168. 10.3389/fcell.2022.974168 36211453 PMC9537699

[56] Gurusaran M Biemans JJ Wood CW Davies OR. Molecular insights into LINC complex architecture through the crystal structure of a luminal trimeric coiled-coil domain of SUN1. Front Cell Dev Biol. (2023) 11:1144277. 10.3389/fcell.2023.1144277 37416798 PMC10320395

[57] Okumura W Tadahira K Kozono T Tamura-Nakano M Sato H Matsui H. Jaw1/LRMP is associated with the maintenance of Golgi ribbon structure. J Biochem. (2023) 173:383–92. 10.1093/jb/mvad004 36689741

[58] Sosa BA Rothballer A Kutay U Schwartz TU. LINC complexes form by binding of three KASH peptides to domain interfaces of trimeric SUN proteins. Cell. (2012) 149:1035–47. 10.1016/j.cell.2012.03.046 22632968 PMC3383001

[59] Jahed Z Shams H Mehrbod M Mofrad MR. Mechanotransduction pathways linking the extracellular matrix to the nucleus. Int Rev Cell Mol Biol. (2014) 310:171–220. 10.1016/B978-0-12-800180-6.00005-0 24725427

[60] Gurusaran M Davies OR. A molecular mechanism for LINC complex branching by structurally diverse SUN-KASH 6:6 assemblies. Elife. (2021) 10:e60175. 10.7554/eLife.60175 33393904 PMC7800377

[61] Murray-Nerger LA Cristea IM. Lamin post-translational modifications: emerging toggles of nuclear organization and function. Trends Biochem Sci. (2021) 46:832–47. 10.1016/j.tibs.2021.05.007 34148760 PMC8793286

[62] Dechat T Pfleghaar K Sengupta K Shimi T Shumaker DK Solimando L. Nuclear lamins: major factors in the structural organization and function of the nucleus and chromatin. Genes Dev. (2008) 22:832–53. 10.1101/gad.1652708 18381888 PMC2732390

[63] Ho CY Jaalouk DE Vartiainen MK Lammerding J. Lamin A/C and emerin regulate MKL1-SRF activity by modulating actin dynamics. Nature. (2013) 497:507–11. 10.1038/nature12105 23644458 PMC3666313

[64] Kong W Wu Z Yang M Zuo X Yin G Chen W. LMNB2 is a prognostic biomarker and correlated with immune infiltrates in hepatocellular carcinoma. IUBMB Life. (2020) 72:2672–85. 10.1002/iub.2408 33211382

[65] Aljada A Doria J Saleh AM Al-Matar SH AlGabbani S Shamsa HB. Altered Lamin A/C splice variant expression as a possible diagnostic marker in breast cancer. Cell Oncol. (2016) 39:161–74. 10.1007/s13402-015-0265-1 26732077 PMC13001873

[66] Burla R La Torre M Saggio I. Mammalian telomeres and their partnership with lamins. Nucleus. (2016) 7:187–202. 10.1080/19491034.2016.1179409 27116558 PMC4916877

[67] Jain N Iyer KV Kumar A Shivashankar GV. Cell geometric constraints induce modular gene-expression patterns via redistribution of HDAC3 regulated by actomyosin contractility. Proc Natl Acad Sci USA. (2013) 110:11349–54. 10.1073/pnas.1300801110 23798429 PMC3710882

[68] Qiu H Sun Y Wang X Gong T Su J Shen J. Lamin A/C deficiency-mediated ROS elevation contributes to pathogenic phenotypes of dilated cardiomyopathy in iPSC model. Nat Commun. (2024) 15:7000. 10.1038/s41467-024-51318-5 39143095 PMC11324749

[69] Danielsson BE George Abraham B Mäntylä E Cabe JI Mayer CR Rekonen A. Nuclear lamina strain states revealed by intermolecular force biosensor. Nat Commun. (2023) 14:3867. 10.1038/s41467-023-39563-6 37391402 PMC10313699

[70] Wu Y Song Y Soto J Hoffman T Lin X Zhang A. Viscoelastic extracellular matrix enhances epigenetic remodeling and cellular plasticity. Nat Commun. (2025) 16:4054. 10.1038/s41467-025-59190-7 40307238 PMC12043949

[71] Bronshtein I Kepten E Kanter I Berezin S Lindner M Redwood AB. Loss of lamin A function increases chromatin dynamics in the nuclear interior. Nat Commun. (2015) 6:8044. 10.1038/ncomms9044 26299252 PMC4560783

[72] Wang Y Ruf S Wang L Heimerl T Bange G Groeger S. The dual roles of lamin A/C in macrophage mechanotransduction. Cell Prolif. (2025) 58:e13794. 10.1111/cpr.13794 39710429 PMC12099221

[73] Chang L Li M Shao S Li C Ai S Xue B. Nuclear peripheral chromatin-lamin B1 interaction is required for global integrity of chromatin architecture and dynamics in human cells. Protein Cell. (2022) 13:258–80. 10.1007/s13238-020-00794-8 33155082 PMC8934373

[74] Ji JY Lee RT Vergnes L Fong LG Stewart CL Reue K. Cell nuclei spin in the absence of lamin b1. J Biol Chem. (2007) 282:20015–26. 10.1074/jbc.M611094200 17488709

[75] Vergnes L Péterfy M Bergo MO Young SG Reue K. Lamin B1 is required for mouse development and nuclear integrity. Proc Natl Acad Sci USA. (2004) 101:10428–33. 10.1073/pnas.0401424101 15232008 PMC478588

[76] Retureau R Foloppe N Elbahnsi A Oguey C Hartmann B. A dynamic view of DNA structure within the nucleosome: biological implications. J Struct Biol. (2020) 211:107511. 10.1016/j.jsb.2020.107511 32311461

[77] Strahl BD Allis CD. The language of covalent histone modifications. Nature. (2000) 403:41–5. 10.1038/47412 10638745

[78] Huynh MT Yadav SP Reese JC Lee T-H. Nucleosome dynamics during transcription elongation. ACS Chem Biol. (2020) 15:3133–42. 10.1021/acschembio.0c00617 33263994 PMC7749077

[79] Li W Hu J Song F Yu J Peng X Zhang S. Structural basis for linker histone H5-nucleosome binding and chromatin fiber compaction. Cell Res. (2024) 34:707–24. 10.1038/s41422-024-01009-z 39103524 PMC11442585

[80] Li Y Zhang H Li X Wu W Zhu P. Cryo-ET study from in vitro to in vivo revealed a general folding mode of chromatin with two-start helical architecture. Cell Rep. (2023) 42:113134. 10.1016/j.celrep.2023.113134 37708029

[81] Swygert SG Lin D Portillo-Ledesma S Lin PY Hunt DR Kao CF. Local chromatin fiber folding represses transcription and loop extrusion in quiescent cells. Elife. (2021) 10:e72062. 10.7554/eLife.72062 34734806 PMC8598167

[82] Coux R Owens NDL Navarro P. Chromatin accessibility and transcription factor binding through the perspective of mitosis. Transcription. (2020) 11:236–40. 10.1080/21541264.2020.1825907 33054514 PMC7714440

[83] Dixon JR Selvaraj S Yue F Kim A Li Y Shen Y. Topological domains in mammalian genomes identified by analysis of chromatin interactions. Nature. (2012) 485:376–80. 10.1038/nature11082 22495300 PMC3356448

[84] Zhang Y Wu Q. CCCTC-binding factor N-terminal domain regulates clustered protocadherin gene expression by enhancing cohesin processivity. J Biol Chem. (2025) 301:108337. 10.1016/j.jbc.2025.108337 39988079 PMC11968269

[85] Ding T Fu S Zhang X Yang F Zhang J Xu H. Inter3D: capture of TAD reorganization endows variant patterns of gene transcription. Genomics Proteomics Bioinformatics. (2024) 22:qzae034. 10.1093/gpbjnl/qzae034 39394698 PMC12016567

[86] Meng L Sheong FK Luo Q. Linking DNA-packing density distribution and TAD boundary locations. Proc Natl Acad Sci USA. (2025) 122:e1876511174. 10.1073/pnas.2418456122 39999165 PMC11892626

[87] Huo X Ji L Zhang Y Lv P Cao X Wang Q. The nuclear matrix protein SAFB cooperates with major satellite RNAs to stabilize heterochromatin architecture partially through phase separation. Mol Cell. (2020) 77:368–83. 10.1016/j.molcel.2019.10.001 31677973

[88] Fukuda K Shimura C Shinkai Y. H3K27me3 and the PRC1-H2AK119ub pathway cooperatively maintain heterochromatin and transcriptional silencing after the loss of H3K9 methylation. Epigenetics Chromatin. (2025) 18:26. 10.1186/s13072-025-00589-3 40312364 PMC12046855

[89] Rowley MJ Corces VG. Organizational principles of 3D genome architecture. Nat Rev Genet. (2018) 19:789–800. 10.1038/s41576-018-0060-8 30367165 PMC6312108

[90] Kureel SK Maroto R Davis K Sheetz M. Cellular mechanical memory: a potential tool for mesenchymal stem cell-based therapy. Stem Cell Res Ther. (2025) 16:159. 10.1186/s13287-025-04249-x 40165288 PMC11960036

[91] Tajik A Zhang Y Wei F Sun J Jia Q Zhou W. Transcription upregulation via force-induced direct stretching of chromatin. Nat Mater. (2016) 15:1287–96. 10.1038/nmat4729 27548707 PMC5121013

[92] Chen Y Xu R Zhou S Zhao C Hu Z Hua Y. Mechanical strain treatment improves nuclear transfer reprogramming efficiency by enhancing chromatin accessibility. Stem Cell Rep. (2023) 18:807–16. 10.1016/j.stemcr.2023.02.007 36963387 PMC10147550

[93] Sia Y Pan H Chen K Chen Z. Structural insights into chromatin remodeling by ISWI during active ATP hydrolysis. Science. (2025) 388:eadu5654. 10.1126/science.adu5654 40179160

[94] Yan L Wu H Li X Gao N Chen Z. Structures of the ISWI-nucleosome complex reveal a conserved mechanism of chromatin remodeling. Nat Struct Mol Biol. (2019) 26:258–66. 10.1038/s41594-019-0199-9 30872815

[95] Osmanagic-Myers S Dechat T Foisner R. Lamins at the crossroads of mechanosignaling. Genes Dev. (2015) 29:225–37. 10.1101/gad.255968.114 25644599 PMC4318140

